# Integrated Profiling Identifies *PLOD3* as a Potential Prognostic and Immunotherapy Relevant Biomarker in Colorectal Cancer

**DOI:** 10.3389/fimmu.2021.722807

**Published:** 2021-09-27

**Authors:** Junhong Shi, Meiyu Bao, Weifeng Wang, Xuan Wu, Yueying Li, Changdong Zhao, Weiwei Liu

**Affiliations:** ^1^ Department of Laboratory Medicine and Central Laboratory, Shanghai Tenth People’s Hospital, Tongji University School of Medicine, Shanghai, China; ^2^ Department of Central Laboratory, Shanghai Tenth People’s Hospital, Tongji University, Shanghai, China; ^3^ Department of Gastroenterology, Second People’s Hospital of Lianyungang City, Lianyungang, China; ^4^ Department of Laboratory Medicine, Shanghai Skin Disease Hospital, Tongji University School of Medicine, Shanghai, China

**Keywords:** Colorectal cancer, *PLOD3*, biomarker, prognosis, immune therapy

## Abstract

Procollagen-Lysine,2-Oxoglutarate 5-Dioxygenase 3 (*PLOD3*) is related to a variety of human diseases. However, its function in Colorectal cancer (CRC) remains uncertain. *PLOD3* expression was analyzed using The Cancer Genome Atlas (TCGA) pan-cancer data. DAVID was used for enrichment analysis of *PLOD3*-related genes. The correlation between *PLOD3* expression and immune cell infiltration was evaluated. Four expression profile datasets (GSE17536, GSE39582, GSE74602, and GSE113513) from Gene Expression Omnibus, and two proteomic datasets were used as validation cohorts for assessing the diagnostic and prognostic value of *PLOD3* in CRC. What’s more, we performed immunohistochemistry (IHC) staining for PLOD3 in 160 paired CRC specimens and corresponding adjacent non-tumor tissues. *PLOD3* was highly expressed in many tumors including CRC. *PLOD3* was upregulated in advanced stage CRCs, and high *PLOD3* expression was associated with poor survival. High *PLOD3* expression was associated with low levels of B cells, CD4^+^ T cells, M1 macrophages, CD8^+^ T cells, and multiple immunerelated characteristics. In addition, the high *PLOD3* expression group had a higher TIDE score and a lower tumor mutation burden and microsatellite instability, indicating that patients with high *PLOD3* expression may be resistant to immunotherapy. Additional datasets and IHC analysis were used to validate the diagnostic and prognostic value of *PLOD3* at the mRNA and protein levels in CRC. Patients with non-response to immunotherapy showed increased *PLOD3* expression in an immunotherapy treated dataset. *PLOD3* is a potential biomarker for CRC diagnosis and prognosis prediction. CRCs with high *PLOD3* expression may be resistant to immune checkpoint therapy.

## Introduction

Colorectal cancer (CRC) is one of the most common malignant tumors of the digestive system, and its morbidity and mortality rates are high worldwide (1). Despite effective cancer screening measures and modern medicine, CRC remains the leading cause of cancer-related mortality worldwide (2). According to the “Cancer Statistics in China”, the incidence and mortality of CRC have increased in China (3). Therefore, it is important to identify novel diagnostic and prognostic biomarkers and to explore potential relevant targets for the treatment of CRC.

Recently, growing evidence has reported that the elevated deposition of collagen and its cross-linking can worsen tumor progression by promoting cancer cell proliferation, migration, and invasion (4, 5). Collagen deposition and cross-linking are dependent on the hydroxylation of lysine residues, which is mainly catalyzed by procollagen-lysine, 2-oxoglutarate 5-dioxygenase (PLOD). *PLOD3*, a member of PLOD family (6), is a multifunctional enzyme with lysyl hydroxylase, collagen galactosyltransferase, and glucosyltransferase activities (7). Collagens constitute a highly specialized family of extracellular matrix (ECM) proteins that maintain tissue architecture and regulate cellular responses (8, 9). *PLOD3* is localized on chromosome 7q22.1, and its activity is critical for the biosynthesis of type IV and VI collagens (10). *PLOD3* overexpression is correlated with high circulating protein levels in some patients (11) and increasing evidence suggested that PLOD3 is associated with tumorigenesis in various cancer types. *PLOD3* is a novel diagnostic marker for early-stage hepatocellular carcinoma (12), human glioma prognosis (13) and ovarian cancer (14). In addition, PLOD3 interacts with STAT3 immunosuppressive signals, which promotes lung cancer metastasis *via* dysregulated RAS-MAP kinase pathway (15). These results suggested an underlying association between PLOD3 and tumor tumorigenesis as well as antitumor immunity. Several pancancer studies (16–18) published in the last year reminded us to explore the molecular features of *PLOD3* using the high-throughput sequencing data. The aim of this study was to uncover the functional role, as well as the diagnostic and prognostic value of *PLOD3* in CRC.

## Methods

### Patients and Samples

A total of 160 paired paraffin-embedded CRC specimens and corresponding adjacent non-tumor tissues were collected to design a tissue array chip from the Shanghai Tenth People’s Hospital, Tongji University School of Medicine. The study was approved by the Research Ethics Committee of Shanghai Tenth People’s Hospital and carried out in accordance with the ethical standards formulated in the Helsinki Declaration. The related ethical approval code is 2020-KN155-01. Tissue microarray was constructed by 1.5-mm cores.

### Immunohistochemistry

**Immunohistochemistry** (IHC) for PLOD3 was carried out on CRC tissue microarray slides. The slides were first incubated at 60°C for 4 h, deparaffinized in xylene, and then rehydrated in alcohol. After heating in citrate buffer for 23 min, we used 0.3% of hydrogen peroxide (H_2_O_2_) to block endogenous peroxidase activity. Slides were blocked with 3% bovine serum albumin for 30 min and incubated in the anti-PLOD3 antibody (diluted 1:200; ab128698; Abcam) overnight at 4°C. The next day, after 3 washes with PBS, slides were incubated with secondary antibody for 1 h, then we used the 3,3-diaminobenzidine (DAB) kit for visualization, and hematoxylin was used to stain nuclei. After the experiments, the slides were observed by microscope. All stainings were scored based on the staining intensity and extensity of positive cells, the intensity (0=genitive, 1=weak, 2=moderate, and 3=strong) and extensity (0 = 5% or less of cells stained positive; 1 = 5%-25%; 2 = 26% to 50%; 3 = 51% to 75%; and 4 = 75% or more) of tumor staining were evaluated. The positive cell density of each core was counted by two independent investigators blind to clinical outcome and knowledge of the clinicopathological data. The final IHC score was calculated by multiplying the strongest intensity score and the total extensity score (maximum value of 12).

### Data Acquisition and Preprocessing

The Cancer Genome Atlas (TCGA) colon adenocarcinoma (COAD) and rectum adenocarcinoma (READ) data, including gene expression quantified by fragments per kilobase million and clinical information of 51 normal tissues and 638 tumor tissues, were obtained from the UCSC Xena project (http://xena.ucsc.edu/). Four independent validation cohorts (GSE17536, GSE39582, GSE74602, and GSE113513) were obtained from the Gene Expression Omnibus (GEO; https://www.ncbi.nlm.nih.gov/geo). Gene level mutations (Mutect2) of the COAD and READ cohorts were acquired from TCGA data portal (https://portal.gdc.cancer.gov/).

### TIMER Database Analysis

The TIMER (12) database was used to analyze differences in *PLOD3* expression between tumors and normal controls from TCGA data set.

### Comprehensive Tumor Immune Analysis

The pan-cancer immune cell infiltration scores for TCGA were obtained from a previously published study (19). The results were based on CIBERSORT (20) and were used for further analysis. TCGA CRC cancer samples were divided into two groups according to median *PLOD3* expression (high *versus* low), and immune cell infiltration was compared between groups. Tumor Immune Dysfunction and Exclusion (TIDE) algorithm (21) was used to estimate a tide score and the predicted response to immune checkpoint blockade. Immunescore and Stromalscore were calculated *via* “estimate” (22) package.

### Biological Functions of *PLOD3* in CRC

The top 1000 genes showing the highest correlation with *PLOD3* were extracted from the LinkedOmics database (http://www. linkedomics.org) (23). Function annotations were performed to identify potentially involved biological processes and signaling pathways using DAVID (24) 6.8 (https://david.ncifcrf.gov/).

### Mutation Analysis

Tumor mutation analysis was performed using the “maftools” package (25). High and low *PLOD3* expression oncoplots were generated *via* “oncoplot” function, and tumor mutation burden (TMB) was calculated using the “maftools” package. Differentially mutated genes between *PLOD3*-high and low groups were evaluated *via* Fisher’s exact test.

### Validation of the Diagnostic, Prognostic and Therapeutic Value of *PLOD3*

Four independent validation cohorts (GSE17536 (26), GSE39582 (27), GSE74602, and GSE113513) were obtained from the GEO database and used as validation cohorts to determine the diagnostic and prognostic value of *PLOD3*. GSE17536 (177 patients) and GSE39582 (585 patients) with relevant survival information were applied for *PLOD3*’s prognostic value validation; GSE74602 (30 pairs) and GSE113513 (14 pairs) including the CRC tissues and matched adjacent tissues were employed for validating the expression difference of *PLOD3* between tumor and normal tissues. In this analysis, patients were divided into low- and high-*PLOD3* groups according to an optimal *PLOD3* cutoff, which was generated using the association between *PLOD3* and survival data with the survminer package. GSE91061 (28) including 105 immunotherapy-treated samples was used for the validation of *PLOD3*’s therapeutic value. After immunotherapy treatment, samples were classified into the following categories according to the patient’s response: complete response (CR), partial response (PR), stable disease (SD) and progressive disease (PD). Among them, CR and PR are recognized as patients who respond to immunotherapy. SD and PD are recognized as patients who do not respond to immunotherapy. Moreover, the protein levels of PLOD3 in colorectal tumors and normal tissues were assessed using a proteomic dataset (29). Another proteomic dataset (30) was used to validate the prognostic value of PLOD3 according to protein expression levels.

### Statistical Analysis

Differences in variables between groups were tested using the Wilcoxon test or chi-squared test, as appropriate. Kaplan-Meier curves were drawn to estimate the overall survival distribution. The log-rank test was used to analyze the statistical difference in survival curves between two groups. Kruskal-Wallis tests were used for comparing *PLOD3*’s expression difference among more than two comparison groups and Wilcoxon test was used for comparison between two groups. The ROC curve was plotted *via* “pROC” package. All figures and statistical analyses were performed using R software (version 4.0.2; http://www.R-project.org). A value of *p* < 0.05 was considered statistically significant. All statistical tests were two-sided.

## Results

### Pan-Cancer *PLOD3* Expression Analysis

**Figure 1:**
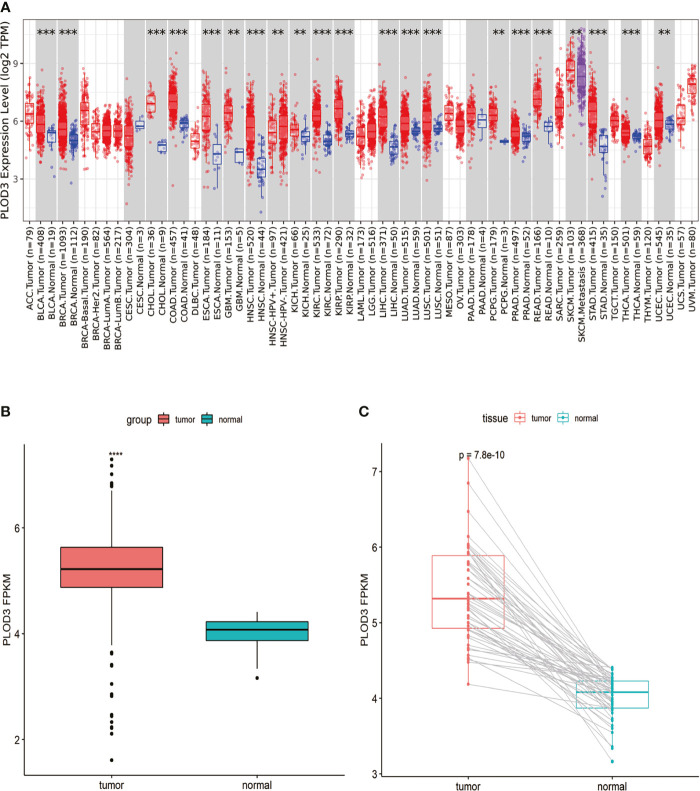
Pan-cancer *PLOD3* expression analysis. **(A)** *PLOD3* expression in tumor and normal tissues from pan-cancer data of The Cancer Genome Atlas (TCGA). **(B)** *PLOD3* expression in tumor and normal tissues from CRC obtained from TCGA. **(C)** *PLOD3* expression in paired CRC tumor and normal tissues from TCGA. Data are expressed as the mean ± SD. **p < 0.01, ***p < 0.001, ****p < 0.0001.

Analysis of *PLOD3* using the TIMER2 database showed that *PLOD3* expression was higher in 19 TCGA tumors than in the corresponding normal tissues, including bladder urothelial carcinoma, breast invasive carcinoma, cholangiocarcinoma, COAD, esophageal carcinoma, Glioblastoma multiforme, head and neck squamous cell carcinoma, Kidney Chromophobe, kidney renal clear cell carcinoma, kidney renal papillary cell carcinoma, Liver hepatocellular carcinoma, lung adenocarcinoma, lung squamous cell carcinoma, Pheochromocytoma and Paraganglioma, Prostate adenocarcinoma, READ, Skin Cutaneous Melanoma, Stomach adenocarcinoma, and Uterine Corpus Endometrial Carcinoma (**Figure 1A**). Particularly, higher *PLOD3* expression was observed in TCGA COAD and READ cohorts, separately and collectively, compared with the adjacent normal tissues (**Figures 1B, C**), suggesting that *PLOD3* plays a role in the pathogenesis of CRC.

### Correlations Between *PLOD3* Expression and Clinical Parameters in CRC Patients

**Figure 2:**
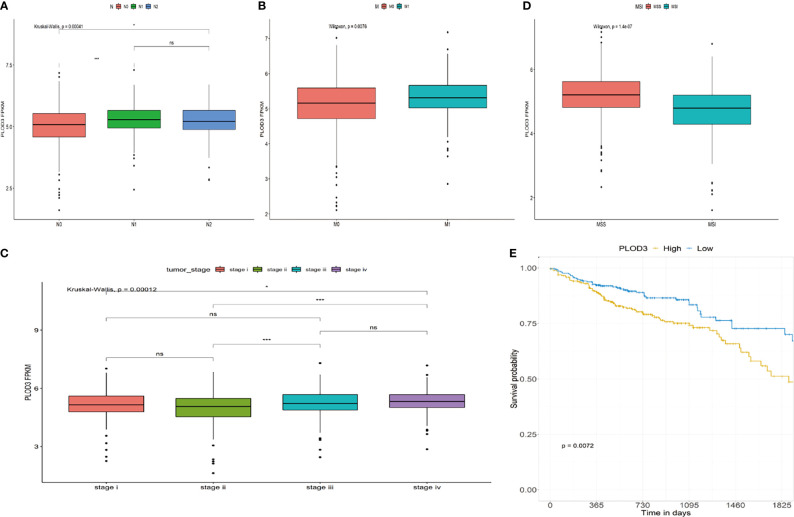
Association of *PLOD3* expression with clinicopathological characteristics, including **(A)** CRC N stage, **(B)** CRC M stage, **(C)** CRC pathologic stage, **(D)** MSI and MSS, and **(E)** overall survival (OS). *p < 0.05, ***p < 0.001, ns, No Significance.

The role of *PLOD3* in CRC remains unclear. Investigating the correlation between *PLOD3* expression and clinical features may clarify the function of *PLOD3* in the progression of CRC. In this study, we examined the relationship between *PLOD3* expression and the clinical parameters of CRC using TCGA cohort. The results showed that *PLOD3* expression differed significantly according to tumor N stage, M stage, clinical stage, and microsatellite instability (MSI) status. Increased N, M, and clinical stages were associated with increased *PLOD3* expression (all *p* < 0.05), suggesting that *PLOD3* may be a poor prognostic factor (**Figures 2A–C**). The microsatellite stable (MSS) group also showed higher *PLOD3* expression (**Figure 2D**). Survival analysis using *PLOD3* median expression as the cut-off value showed that CRCs with higher expression of *PLOD3* had a worse prognosis than those with lower expression (log rank *p* < 0.01) (**Figure 2E**).

### Gene Function Annotation and Pathway Analysis

**Figure 3:**
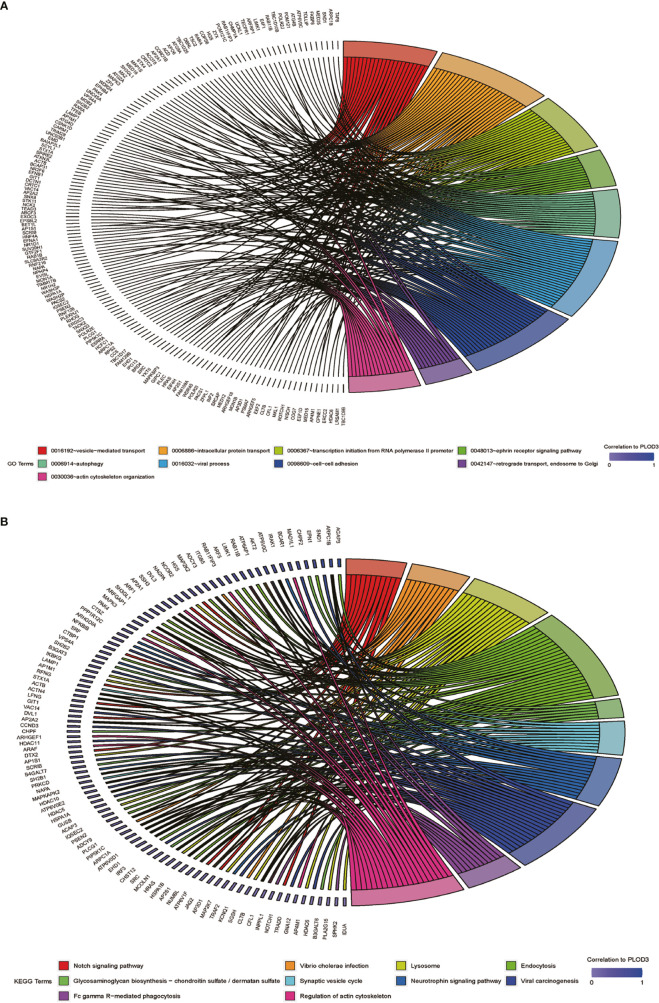
**(A)** GO BP pathway analysis of PLOD3 related genes in CRC. **(B)** KEGG pathway analysis of PLOD3 related genes in CRC.

After determining the prognostic value of *PLOD3* in CRC, we next explored the biological functions associated with *PLOD3*. GO and KEGG enrichment analyses were performed, and the top GO terms and signaling pathways are shown in **Figures 3A, B**. *PLOD3* gene expression was associated with many biological processes, such as vesicle-mediated transport, ephrin receptor signaling pathway, and autophagy. *PLOD3* was also associated with Notch signaling, neurotrophin signaling, and glycosaminoglycan biosynthesis.

### Differences in Genomic Mutation Profiles and TMB Between Different *PLOD3* Groups

**Figure 4:**
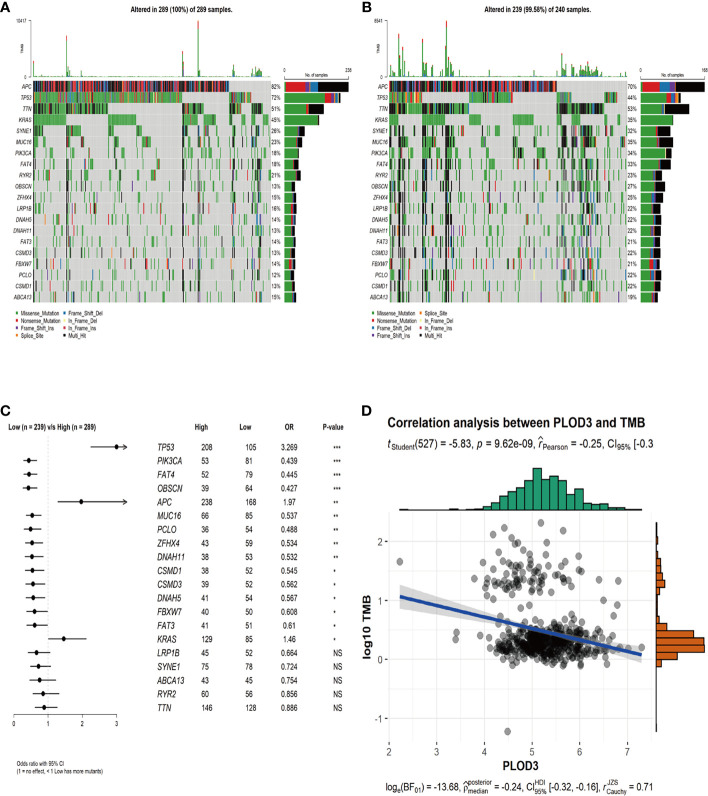
Genomic mutation profiles and TMB characteristics of *PLOD3*-high and *PLOD3*-low groups. **(A)** Distribution of top 20 frequently mutated genes in *PLOD3*-high TCGA-CRC subgroups. The upper bar plot shows the TMB for each patient, and the left bar plot indicates the gene mutation frequency in different risk groups. **(B)** Distribution of top 20 frequently mutated genes in *PLOD3*-low TCGA-CRC subgroups. The upper bar plot shows the TMB for each patient, and the left bar plot indicates the gene mutation frequency in different risk groups. **(C)** Differentially mutated genes between *PLOD3*-high and low groups. The p values were calculated by Fisher’s exact test. *p < 0.05; **p < 0.01; ***p < 0.001, NS, No Significance. **(D)** Correlation analysis of TMB and *PLOD3* in TCGA-CRC cohort.

To examine the relationship between *PLOD3* and mutation profiles in CRC, tumor mutations were compared between *PLOD3* high and low groups. We chose the top 20 mutation genes in the whole CRC cohort and compared the mutation frequency difference between *PLOD3* high (**Figure 4A**) and low (**Figure 4B**) groups using the Fisher’s test. Furthermore, we visualized these mutation genes in a forest (**Figure 4C**). *TP53*, *APC*, and *KRAS*, were significantly mutated in the *PLOD3*-high group, whereas *PIK3CA*, *FAT4*, and *OBSCN* were specifically mutated in the low-expression group. In addition, a significant (*p* < 0.001) negative correlation was observed between *PLOD3* and TMB (**Figure 4D**).

### *PLOD3* and the Immune Microenvironment in CRC

**Figure 5:**
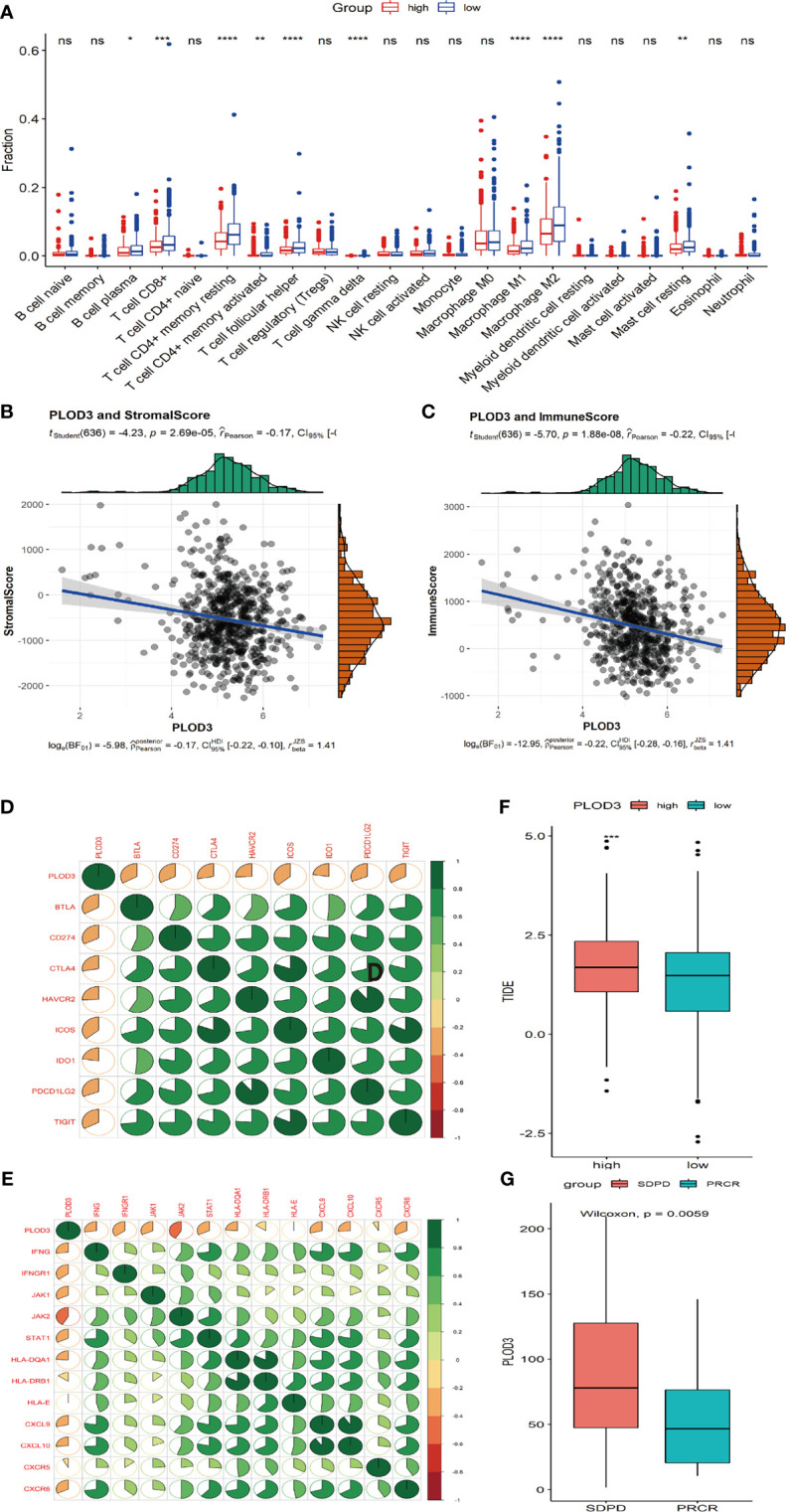
Correlation analysis of immune cell infiltration and *PLOD3* in CRC. **(A)** Immune cell infiltration levels in high and low *PLOD3* expression groups from TCGA-CRC cohort. **(B)** Correlation analysis of StromalScore and *PLOD3* in TCGA-CRC cohort; **(C)** Correlation analysis of ImmueScore and *PLOD3* in TCGA-CRC cohort. **(D)** Correlation between *PLOD3* and immune checkpoint levels; green represents positive correlation, red represents negative correlation; color intensity is positively related with the strength of the correlation. **(E)** Correlation between PLOD3 and immune related genes; green represents positive correlation, red represents negative correlation; color intensity is positively related with the strength of the correlation. **(F)** Boxplot shows the TIDE score for high and low PLOD3 expression groups. Data are expressed as the mean ± SD. * *p* < 0.05, ** *p* < 0.01, ***p < 0.001, **** *p* < 0.0001. ns, not significant. **(G)** Boxplot shows the PLOD3 expression for immunotherapy response (CR and PR) and non-response groups (SD and PD). CR, complete response; PR, partial response; SD, stable disease; PD, progressive disease.

Next, we analyzed the immune cell infiltration difference between *PLOD3* high and low groups. The infiltration scores of B cell plasma, T cell CD8+, T cell CD4 memory resting, T cell CD4 memory activated, T cell follicular helper, T cell gamma delta, and macrophage M1 were higher in the *PLOD3*-low cohort than in the *PLOD3*-high cohort (**Figure 5A**). *PLOD3* was significantly negatively correlated with StromalScore (**Figure 5B**) and immuneScore (**Figure 5C**). In addition, *PLOD3* was significantly negatively correlated with multiple immune checkpoints (**Figure 5D**) and many other immune related genes, such as antigen-presentation, chemokines, interferons, and T cell inflamed genes (**Figure 5E**). What’s more, a significantly higher TIDE score was observed in *PLOD3*-high group (**Figure 5F**). In an immunotherapy-treated cohort, patients showed non-response to immunotherapy presented with higher *PLOD3* expression (**Figure 5G**). Given that higher *PLOD3* expression was associated with lower immunescore, infiltration of multiple immune cells and many immune-related genes, TMB, and MSI score, consistent with the higher TIDE score, we speculated that CRC patients with high *PLOD3* expression may be resistant to immunotherapy, which was justified in an immunotherapy-treated cohort.

### Validation of the Diagnostic and Prognostic Value of *PLOD3*

**Figure 6:**
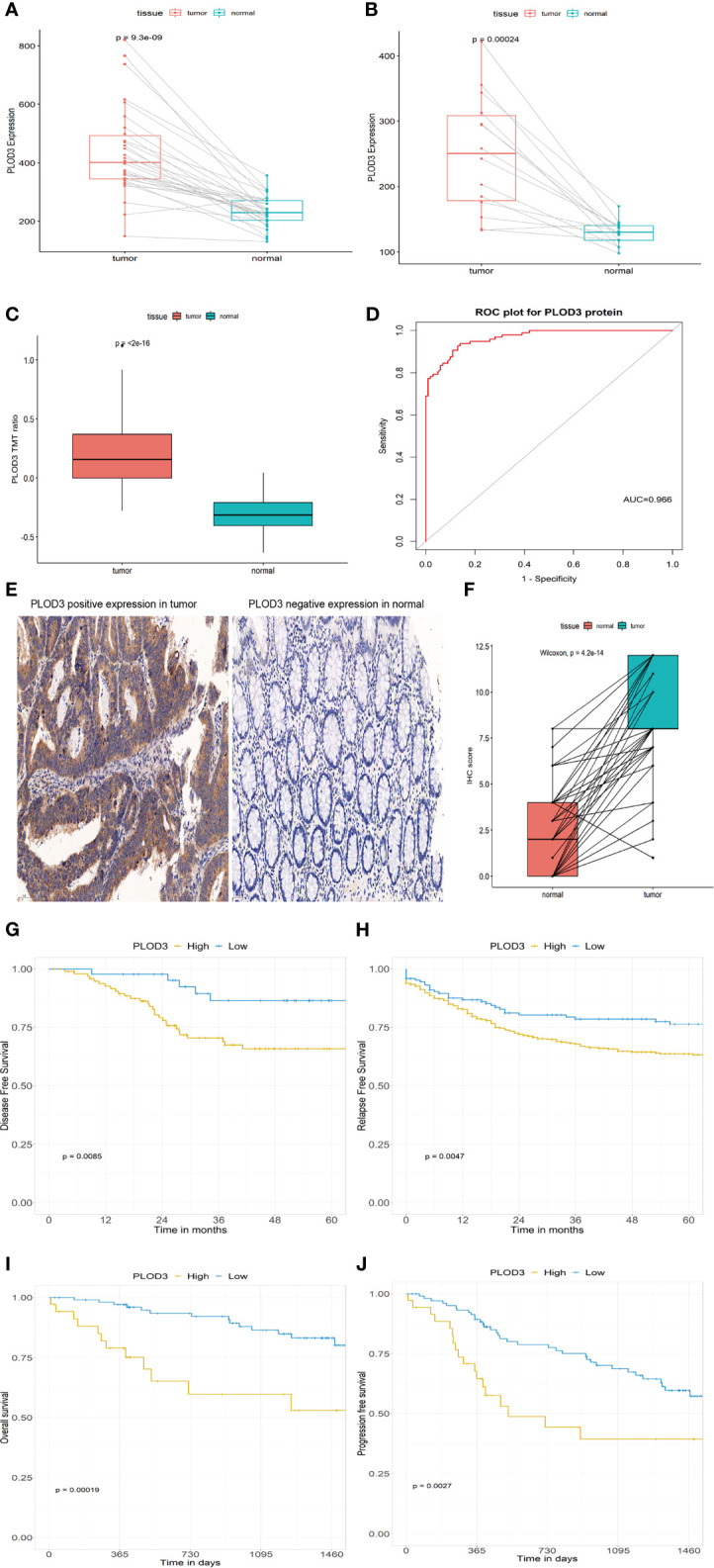
*PLOD3* expression in CRC and normal tissues from the GSE74602 **(A)** and GSE113513 **(B)** cohorts; *PLOD3* protein expression difference in CRC and normal tissues and TMT ratio means the protein abundance by TMT-based quantitation **(C)** and ROC curve of *PLOD3* protein for discriminating tumor and normal tissues in proteome dataset **(D)**; **(E)** Representative microphotographs of PLOD3 immunohistochemical staining in CRC tissue and adjacent normal tissue by IHC. Scale bar = 50 μm. **(F)** IHC scores in CRC tissues and adjacent tissues. Prognostic value of *PLOD3* in the GSE17536 **(G)** and GSE39582 **(H)** cohorts; Prognostic value of *PLOD3* in the proteome dataset **(I, J)**.

Two GEO datasets (GSE74602 and GSE113513) were used to validate the diagnostic value of *PLOD3* using paired tumor and normal samples. *PLOD3* expression was significantly higher in tumor than in normal samples (**Figures 6A, B**). PLOD3 protein expression was higher in CRC than normal tissues (**Figure 6C**). **Figure 6D** showed PLOD3 protein presented with good diagnostic value between CRC and normal tissues. To further confirm the diagnostic value of PLOD3 in CRC patients, the expression of PLOD3 were analyzed by TMA-based IHC. we compared 160 CRC tumor tissues with paired adjacent normal tissues in a microarray, the representative IHC images of positive PLOD3 expression in tumor tissue and negative PLOD3 expression in normal tissue were shown (**Figure 6E**). Grossly, PLOD3 was overexpressed in tumor parts comparing to normal specimens (**Figure 6F**, p-value = 4.2e-14).

GSE17536 and GSE39582 were used to validate the prognostic value of *PLOD3*. In the GSE17536 dataset, patients with higher *PLOD3* expression had a worse disease-free survival (**Figure 6G**). In GSE39582, patients with higher *PLOD3* had a worse progression-free survival (**Figure 6H**). The proteomic dataset generated by Li et al. (30) was used to validate the prognostic value of PLOD3 protein expression, and the results revealed that patients with higher PLOD3 protein expression had a worse overall survival and progression-free survival (**Figures 6I, J**). Taken together, the results indicate that *PLOD3* is a promising biomarker for the diagnosis, prognosis, and treatment of CRC.

## Discussion

PLOD3, a collagen biosynthesis-related protein, was reported to contributes to carcinogenesis of HCC (12), glioma (13), ovarian cancer (14, 31), and lung cancer (15). However, the function of *PLOD3* in CRC remains to be elucidated. In the current study, we found that *PLOD3* was expressed at high levels in CRC tissues, and patients with higher *PLOD3* expression had worse survival. These results were validated at the mRNA and protein levels using additional datasets. CRCs with higher *PLOD3* expression showed a lower TMB, a higher TIDE score, and patients with MSS tended to have higher *PLOD3* expression, suggesting that these patients could present with immunotherapy resistance. An immunotherapy-treated cohort was enrolled to validate *PLOD3*’s predictive role for immunotherapy response.

*PLODs* are mainly regulated at the transcriptional level. For instance, hypoxia-induced factor-1 activates *PLOD1* in breast cancer, and to a great extent, activates *PLOD2* in cancer development (32). *PLOD1* was found to be directly regulated by miR-140-5p and abnormally expressed *PLOD1* induced cancer aggressiveness in bladder cancer (33). Unlike *PLOD1* and *PLOD2*, the regulation of *PLOD3* in CRC is poorly understood. Data mining using TCGA datasets showed that *PLOD3* was overexpressed in 19 types of tumor tissues compared with normal tissues, indicating that *PLOD3* may serve as a novel biomarker in cancer. Increased *PLOD3* expression in CRC tumor tissues at the mRNA and protein levels indicated the potential diagnostic value of *PLOD3*. The prognostic value of *PLOD3* was validated in other datasets, confirming its prognostic potential.

In the present study, patients with advanced stage CRC showed higher *PLOD3* expression, indicating that *PLOD3* may be associated with metastasis. *PLODs* are implicated in metastasis because of their role in regulating collagen biosynthesis (34, 35). Collagens provide the scaffold for ECM assembly and are considered “highways” for cancer cell migration (36). *MicroRNA-663a* targets the 3′ untranslated region of *PLOD3* and decreases its expression, resulting in decreased accumulation of extracellular collagen (37). GO and KEGG enrichment analysis of *PLOD3*-related genes identified many associated pathways, such as the Notch signaling, neurotrophin signaling, and glycosaminoglycan biosynthesis pathways. Neurotrophin expression is associated with poor prognosis in cutaneous melanoma. The Notch signaling pathway is activated in CRC and other cancer types (38, 39) and could be related to poor-prognosis subtypes and metastasis in CRC (40), which may explain the association of *PLOD3* with poor prognosis. Some studies revealed the worse prognostic value of *TP53 (* 41) and *KRAS* for CRCs (42). Higher mutation frequency of *TP53* and *KRAS* were observed in high-*PLOD3* group, which may account for the worse survival for this group. In contrast, *FAT4* was highly mutated in the *PLOD3*-low group and Zhuang et al. has demonstrated the better prognostic value of *FAT4* in CRC (43).

Immune cells play a role in the regulation of tumor cell behavior (44, 45), and accumulating evidence supports their significance in predicting outcomes and therapeutic efficacy in many cancer types (45, 46). In the present study, the infiltration levels of CD8-positive T cells and M1 macrophages were significantly lower in the *PLOD3*-high CRC group. *OBSCN (* 47) was significantly associated favorable prognosis, immune-hot subtype and potentially better immunotherapeutic efficacy, which was consistent with more immune infiltration in *PLOD3*-low group. Moreover, negative correlations were observed between *PLOD3* expression and multiple immune related genes (48, 49), suggesting that *PLOD3* plays a negative role in regulating tumor immunology. Several indicators for immunotherapy response have been identified in CRC, such as TMB (50, 51) and MSI (52, 53) status. Remarkably, the expression of the *PLOD3* was negatively correlated with immuneScore and a previous study (54) in CRC indicated that CRC patients with a lower immuneScore had a poor overall survival. CRCs with higher *PLOD3* showed a lower ImmuneScore, TMB, a higher TIDE score, and patients with MSS tended to have higher *PLOD3* expression, suggesting the potential for immunotherapy resistance in CRC patients, which was validated in an immunotherapy treated cohort.

The fecal occult blood test methods are more easily accepted by patients, currently but they often suffered various interfering factors with some causes of false-negative, false-positive results, and low sensitivity rates for detecting colon polyps (55). Therefore, early, non-invasive, specific, and sensitive biomarkers are still required for screening strategies in colorectal cancer. We firstly reported the diagnostic and prognostic value of *PLOD3* in CRC and validated our finding in other transcriptome and proteome datasets. Given *PLOD3*’s good discriminating ability and its prognostic value, *PLOD3* was a promising biomarker for CRC.

Finally, we discovered, for the first time, the effect of *PLOD3* on CRC. We performed a comprehensive analysis to evaluate the functional role of *PLOD3* in CRC. The results suggest that *PLOD3* is a promising biomarker for the diagnosis and prognosis of CRC. In addition, the study evaluated the performance of *PLOD3* as a potential indicator for immunotherapy in CRC patients. These findings can facilitate the personalized treatment of CRC patients. However, the study had several limitations: The PLOD3’s predictive role for immunotherapy was not validated in an immunotherapy-treated CRC cohort and further investigations are needed in the future.

In summary, *PLOD3* was identified as a promising biomarker for the diagnosis and prognosis prediction of CRC, and it could be valuable for the design of individualized treatment strategies for CRC patients.

## Data Availability Statement

The datasets analyzed during this study are available in the TCGA database (https://portal.gdc.cancer.gov) (TCGA-COAD and TCGA-READ), GEO database (https://www.ncbi.nlm.nih.gov/geo) (GSE17536, GSE39582, GSE74602, and GSE113513).

## Author Contributions

JS did bioinformatic analysis and wrote the manuscript. JS, XW, and YL collected the data. JS, MB, WW, CZ, and WL designed the research, organized the calculations. All authors contributed to the article and approved the submitted version.

## Funding

This study was supported by grant from the Outstanding academic leaders plan of Shanghai (Grant No. 2018BR07) and “Qinglan Inheritance Project” Fund Project of the Second People’s Hospital of Lianyungang City.

## Conflict of Interest

The authors declare that the research was conducted in the absence of any commercial or financial relationships that could be construed as a potential conflict of interest.

## Publisher’s Note

All claims expressed in this article are solely those of the authors and do not necessarily represent those of their affiliated organizations, or those of the publisher, the editors and the reviewers. Any product that may be evaluated in this article, or claim that may be made by its manufacturer, is not guaranteed or endorsed by the publisher.
